# Characterising the Transcriptomic Response to Interferon and Infection in European Domestic Ferret Respiratory Tissues Using Long‐Read RNA Sequencing

**DOI:** 10.1111/imm.70042

**Published:** 2025-10-07

**Authors:** Rubaiyea Farrukee, Jessie J.‐Y. Chang, Jianshu Zhang, James B. Barnes, Shu Xin Zhang, Sher Maine Tan, Patrick C. Reading, Lachlan J. M. Coin

**Affiliations:** ^1^ Department of Microbiology and Immunology University of Melbourne, The Peter Doherty Institute for Infection and Immunity Victoria Australia; ^2^ WHO Collaborating Centre for Reference and Research on Influenza Victorian Infectious Diseases Reference Laboratory at the Peter Doherty Institute for Infection and Immunity Melbourne Victoria Australia

## Abstract

The European domestic ferret ( *Mustela putorius furo* ) is considered the gold standard small animal model for studying human and avian influenza virus infections. However, experimental characterisation of the transcriptomic response to interferon (IFN) stimulation and/or influenza virus infection has been limited, particularly in defining the induction of interferon‐stimulated genes (ISGs), with most being computationally predicted. In this study, we present a comprehensive transcriptome‐wide assessment of the ferret transcriptome following IFN‐α treatment of a ferret lung (FRL) cell line, as well as in nasal turbinates from influenza A virus (IAV)‐infected ferrets using long‐read RNA sequencing. We have identified a panel of ferret genes orthologous to human ISGs that are upregulated both in response to IFN‐α stimulation in vitro and IAV infection in vivo. We have also identified novel IFN‐stimulated genes and transcripts. Furthermore, we observed elongation of the poly(A) tails of genes in the *ribosome* and *Coronavirus Disease‐19* pathways in response to IFN‐α treatment in vitro*,* suggesting a relationship between poly(A) elongation and the antiviral responses of the host. These results illuminate the dynamics of the transcriptional innate immune response of the domestic ferret and provide an important resource for better utilising ferrets as a small animal model to study influenza virus infections.

## Graphical abstract

We utilised long‐read Nanopore sequencing to characterise the transcriptome of ferret cell lines stimulated with IFN‐α as well as nasal turbinates from ferrets infected with influenza A virus. We identified novel genes and isoforms and observed elongation of poly(A) tails in the ribosome and *Coronavirus Disease‐19* pathways in response to IFN‐α treatment.

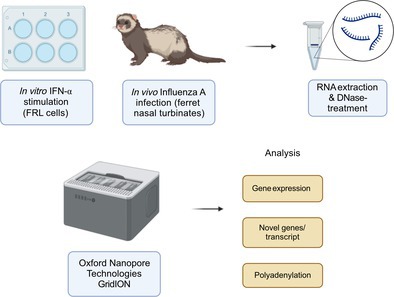

## 1 Introduction

Ferrets are widely regarded as the gold‐standard animal model for studying influenza virus infections in vivo and offer a number of advantages over conventional mouse models [1]. Ferrets share similarities with humans in their lung architecture and in the distribution of particular sialic acid receptors in their airways and can therefore be infected with a wide range of human influenza A viruses (IAVs) without the need for prior host adaptation [1, 2]. Also, unlike mice, ferrets show clinical signs of infection (sneezing, fever, lethargy, etc.) and can transmit virus between animals via direct contact or by aerosols, making them an ideal model for measuring the impact of therapeutic interventions on clinical outcomes of IAV infection and transmission. In addition, ferrets are outbred animals that show a high degree of genetic similarity (> 99% at mitochondrial level) to European minks (*Mustela luterola*), which represent an intermediate reservoir species for avian IAV infections [3]. This was highlighted in the 2022 outbreak of highly pathogenic avian influenza (HPAI) H5N1 virus (Clade 2.3.4.4b) in mink farms in Spain, which resulted in the culling of > 50 000 animals [4]. While previous studies confirm that mink are susceptible to both human and avian IAV [5], few experimental studies have used mink, whereas ferrets are widely used and therefore serve as an ideal surrogate model for mink.

Despite the well‐established use of ferrets to study pathogenesis and immunity to influenza virus, limited tools and reagents are currently available to characterise the immune responses elicited in these animals. Studies examining innate immune responses to virus infection, characterised by the secretion of Types I and III interferons (IFNs) and upregulation of interferon‐stimulated genes (ISGs), are limited and generally rely on qPCR to detect gene expression [6, 7]. In humans and mice, many ISGs are known to mediate antiviral activity against a broad range of viruses, including IAV; however, less is known regarding their role in other species such as ferrets. We recently characterised ferret Mx1 (an ISG which shows 100% sequence identity to European mink Mx1 at the mRNA level), reporting that it differed significantly from human Mx1 in the potency of its antiviral activity against human seasonal IAV [8]. These findings highlight key differences in the function of a particular ISG between humans and ferrets/mink. However, the ferret genome and the transcriptome are poorly annotated on the National Center for Biotechnology Information (NCBI) database, making it difficult to identify ferret orthologs of additional human ISGs with known antiviral activity against IAV [8]. It remains important to characterise ferret orthologues to define similarities and differences between human and ferret ISGs, as such studies have important implications for contextualising the relevance of conclusions drawn from ferret studies to human influenza infections. Improved annotation of the ferret genome and transcriptome, as well as functional validation of key ISGs, would also enhance the utility of the ferret model for evaluating the efficacy of antiviral therapies and vaccines targeting innate immune pathways [9]. Moreover, a better understanding of the ferret ISG repertoire may also reveal host‐specific ISG responses that can shape viral evolutionary trajectories. This knowledge is especially relevant for zoonotic viruses such as avian IAVs, which may undergo adaptive changes in intermediate hosts such as ferrets and mink before acquiring the capacity to infect humans more efficiently. Thus, bridging the current knowledge gap in ferret innate immunity is essential to maximise the utility of this valuable animal model and to advance our understanding of host adaptation of influenza viruses.

Third‐generation sequencing platforms such as Oxford Nanopore Technologies (ONT) allow the full‐length sequencing of RNA transcripts, enabling an enhanced view of the whole transcriptome [10, 11]. The technology is particularly useful for detecting novel transcripts due to accurate elucidation of all possible alternative splicing events on one single strand of RNA, which is arduous and near impossible using short‐read sequencing platforms such as Illumina [12]. Therefore, as most of the ferret transcriptome is still largely predicted, ONT long‐read sequencing provides an efficient method to validate long transcripts with multiple alternative splicing events. This ability extends to visualising trans‐spliced transcripts, which are transcripts derived from two pre‐mRNA molecules during the splicing process [13]. Furthermore, an additional advantage of ONT RNA‐sequencing is its ability to capture the full length of the poly(A) tail on transcripts [12, 13, 14, 15], which is also limited in short‐read sequencing [16]. Poly(A) tails on mRNA are known to be associated with various molecular functions, such as mRNA circularisation, localisation, decay rates, stability and regulation of gene expression [14, 17, 18, 19, 20, 21, 22]. While short‐read sequencing has been previously used to define the ferret transcriptome [23, 24, 25] and the diversity of its B cell receptors [26], to our knowledge, a thorough investigation into the transcriptome and polyadenylome of domestic ferret following IAV infection using long‐read sequencing has not been reported. Thus, in this study, we sought to utilise the advantages of long‐read Nanopore sequencing to comprehensively interrogate the transcriptome of the European domestic ferret ( *Mustela putorius furo* ).

## 2 Results

### 2.1 General Features and Metrics of the Domestic Ferret Transcriptome

**FIGURE 1:**
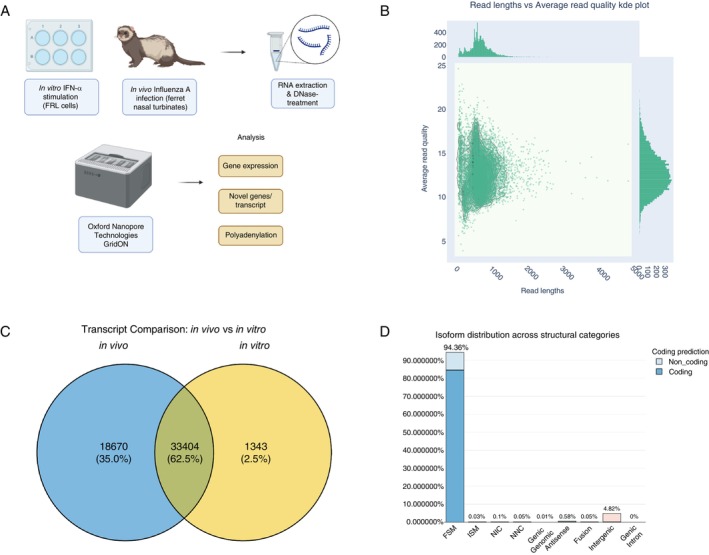
Characterisation of ferret transcripts sequenced for this study. (A) Flowchart of methods and analysis. Briefly, immortalised ferret lung (FRL) cells were cultured in the presence or absence of recombinant ferret IFN‐α (in vitro) and domestic ferrets were mock‐infected or infected via the intranasal route with IAV strain A/Perth/269/2009 (in vivo). Total RNA was extracted from the FRL cells 24 h post‐treatment and nasal turbinate samples were collected from ferrets 6 or 24 h post‐infection (hpi), which were then treated with DNase. The RNA was sequenced on the ONT GridION using the SQK‐PCB111.24 kit. Data analysis steps included gene expression, novel gene/transcript discovery and poly(A) analyses. (B) Scatter kde plots for read lengths and average read quality derived from ONT PCR‐cDNA sequencing. *x*‐axis shows read‐lengths in nucleotides and *y*‐axis shows average read quality. (C) Venn Diagram showing the number of transcripts discovered in the in vivo versus in vitro datasets. (D) *SQANTI3* isoform structures distributions across detected transcripts post‐filtering via *SQANTI3*.

To characterise the ferret transcriptome, samples were derived from ferret lung epithelial (FRL) cells cultured for 24 h in the presence or absence of recombinant ferret IFN‐α, as well as nasal turbinates isolated from ferrets 6 or 24 h post‐infection (hpi) after intranasal infection with PBS (mock) or IAV strain A/Perth/269/2009 (Perth/09, H1N1pdm09) (Figure 1A). Following RNA extraction, samples were sequenced using the ONT PCR‐cDNA Barcoding kit. As the ferret transcriptome is still largely incomplete, we investigated the presence of hypothetical (‘XM’) transcripts (as annotated on NCBI) and unannotated novel transcripts. A total of ~46.3 M passed reads were sequenced and re‐stranded, with an N50 read length of 743 nt and median length quality score of 12.6 (Figure 1B).

To visualise the ferret transcriptome through our long‐read datasets, we used a combination of *Bambu* and *SQANTI3* pipelines. After *Bambu* was used to detect novel transcripts and create a new annotation file, we checked the characteristics of the annotated reads using the *SQANTI3* ‘qc’ function(s). Of the final list of true isoforms, it was evident that more isoform models were able to be detected in the in vivo datasets compared with the in vitro datasets (Figure 1C). Furthermore, most isoforms (~94.36%) were determined to be of the Full‐Splice Match (FSM) class and some were of the intergenic class (~4.82%) (Figure 1D and Data S1, see Section 4 for the definition of transcript model classes). This indicates that most transcripts matched the splice junctions of existing reference transcripts and that some were found between genes, likely due to the previously unannotated novel genes. After final *Bambu* quantification, we observed novel (1414), known (‘NM’: 64, ‘NR’: 0) and hypothetical (‘XM’: 57 475, ‘XR’: 10 942) transcripts and 1120 novel genes. These results highlight that many of the predicted transcripts can indeed be found in vitro and in vivo and the strength of utilising ONT sequencing for novel transcript discovery.

### 2.2 Identification and Regulation of Ferret Genes Orthologous to Human ISGs

**FIGURE 2:**
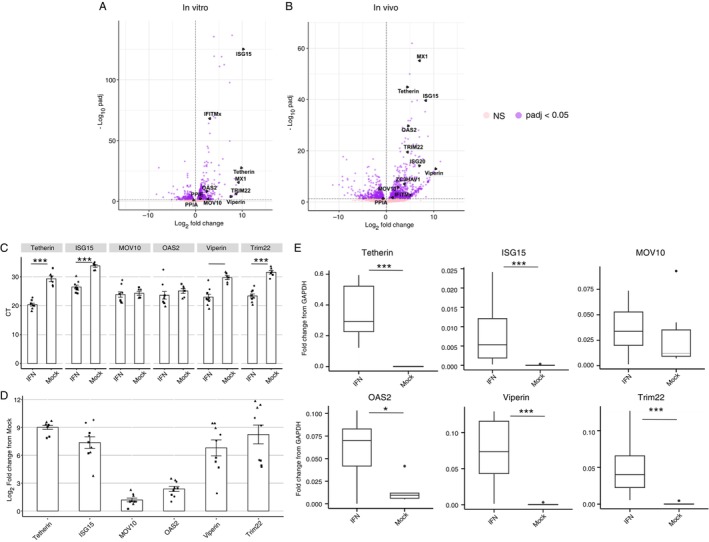
Upregulation of ferret genes orthologous to particular human ISGs in response to IFN treatment (in vitro, 24 h post‐treatment) or IAV infection (in vivo, 24 h post‐infection). Differential expression results in (A) in vitro and (B) in vivo datasets at the gene level. *x* axis represents log_2_ fold changes and the *y* axis shows the ‐log_10_ *p*‐adjusted value (*p*_adj_), with a threshold of *p*_adj_ < 0.05, shown by the dotted horizontal line. (C–E) Six ISGs were validated following analysis of FRL cells cultured for 24 h in the presence or absence of IFN. RT‐qPCR results show (C) raw CT differences between mock‐ and IFN‐treated FRL cells, (D) Log_2_ fold change in expression levels relative to mock controls, calculated using the 2^−ΔΔ*C*T^ method and (E) fold change relative to housekeeping gene GAPDH using the 2^−Δ*C*T^ method. Pooled data from three independent experiments are shown; different symbols are used to denote each experiment. Statistical differences in CT and fold‐change values from mock (C and E) were calculated using a mixed effects model. *p < 0.05, **p < 0.01, ***p < 0.001.

To determine if ferret orthologues of human ISGs with known anti‐IAV activity had been upregulated [7], we performed differential expression analyses to compare mock/control samples to IFN‐α‐treated (in vitro) or IAV‐infected (in vivo) samples. Differential expression analyses revealed 1120 significantly differentially expressed genes (DEGs) between mock‐ and IFN‐α‐treated FRL cells (in vitro, *p*_adj_ < 0.05, Figure 2A). For in vivo datasets, we observed 20 and 1131 DEGs at 6 (Figure S1A, *p*_adj_ < 0.05) and 24 hpi (Figure 2B, *p*_adj_ < 0.05), respectively, compared to mock controls. No ferret ISG orthologues of interest were upregulated at 6 hpi, suggesting that innate immune pathways require additional time for activation and expression of ISGs in IAV‐infected ferrets. However, when examining in vitro and in vivo analyses at 24 h post‐IFN treatment or 24 hpi, respectively, we observed significant upregulation of many ferret ISG and IST orthologues of interest compared to the relevant mock controls (Figures 2A,B and S1B–D). The upregulation of a select group (*n* = 6) of ferret ISGs, which represent orthologues of human ISGs with known anti‐IAV activity, was validated using RT‐qPCR to examine induction in FLR cells cultured for 24 h in the presence or absence of ferret IFN‐α. With the exception of *MOV10*, all ferret ISGs tested were significantly upregulated following IFN treatment (Figure 2E, *p* < 0.01) and certain ISGs such as *Tetherin, ISG15, Viperin* and *TRIM22* were potently upregulated (300–500‐fold compared to mock, Figure 2D). Ferret *OAS2* was moderately upregulated (~8‐fold compared to mock). Our previous study used colony PCR to confirm that the major transcript of ferret *Mx1* produced in FRL cells was XM_004762192.2 [8]. Herein, Nanopore sequencing confirms this result (Figure 2A) and shows that this *Mx1* transcript is also strongly upregulated in the nasal turbinates of ferrets 24 h after IAV infection (Figure 2B). Finally, we note that the trend in fold changes observed in RT‐qPCR data (Figure 2D) correlates well with differential analysis data obtained following Nanopore sequencing (Figure 2A).

**FIGURE 3:**
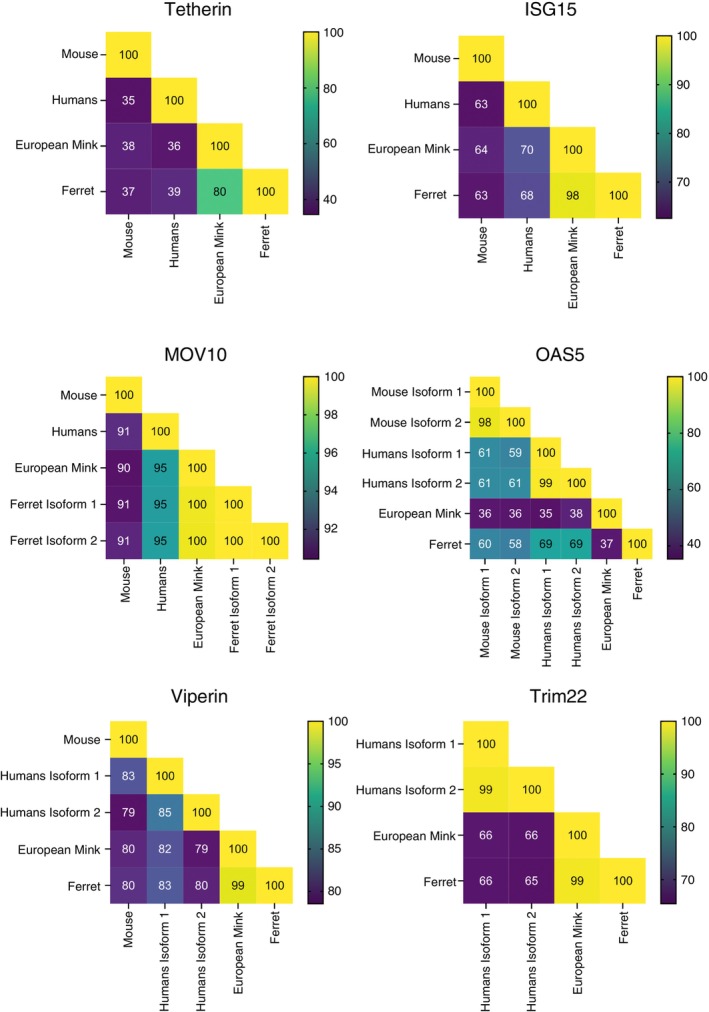
The protein coding sequence (CDS) for a select group of ISGs obtained from NCBI (for humans, mice and European mink ( *Mustela lutreola* )) or from our data set (ferret) was aligned using *MUSCLE*. The percentage identity between each species is represented in a heatmap for each ISG. Of note, mice do not express an orthologue of TRIM22. For European mink, genome annotation remains incomplete and all protein CDS information was derived from predicted transcripts (denoted by XM accession numbers, see Section 4). Moreover, we detected two isoforms of ferret MOV10, with isoform 1 representing the major species in vitro and isoform 2 in vivo. While minor isoforms of other ferret ISGs were detected, their expression levels were very low and they have therefore been excluded from this analysis.

In addition to identifying ferret ISG transcripts that represent orthologues to human ISGs with known anti‐IAV activity, we aimed to determine their similarity between different mammalian species. Therefore, we compared the predicted protein coding sequences of ferret ISGs identified in this study to published sequences of ISGs from humans, mice and European mink, noting that mice represent the small animal model most commonly used to study IAV in vivo (Figure 3). While human and mouse genomes are well annotated, the European mink genome is incomplete and therefore predicted transcript sequences of mink ISGs were used in these analyses. Alignment results indicate that, with the exception of *OAS2*, all ferret ISGs have the highest degree of similarity to European mink. Dissimilarity between ferret and mink *OAS2* is likely artefactual and due to errors in annotation of the mink genome. *MOV10* and *Viperin* were the most conserved ISGs across different species (> 80% similarity in protein sequence), while *Tetherin* showed the highest degree of divergence (Figure 3). In general terms, most ferret ISGs were closer to human than to mouse ISGs.

The human ISGs *Tetherin, ISG15, MOV10, OAS2, Viperin* and *TRIM22* are very well characterised and as such, we know certain regions/sites/motifs are important for their protein localisation, stability, signalling, antiviral activity, post‐translational modifications and so forth [7]. We therefore utilised our alignments (Figure 3) to interrogate whether these sites/regions/motifs were conserved between humans and ferrets, which can provide insights into whether these ISGs have similar function (and therefore similar antiviral activities) across the two species. Our results show that all major sites relevant to protein function and the antiviral activity of *ISG15* and *MOV10* are conserved between humans and ferrets, whereas differences were noted in some major sites between human and ferret *Tetherin, OAS2, Viperin* and *TRIM22* (Table 1). These differences pertain to aspects of protein function such as NFκβ activation or enzyme activity (*Tetherin* and *OAS2*), protein localisation (*TRIM22*) or protein–protein interactions (*Viperin*), highlighting species‐specific differences in ISG structure that could be validated experimentally in future studies.

**TABLE 1:** Degree of conservation in regions/sites needed for protein function, signalling and/or antiviral activity (human amino acid numbering).

Gene	Conserved sites/regions/motifs	Non‐conserved sites/regions/motifs
Tetherin	Sites needed for NFκβ activation (6Y, N65, N92, N123) and homodimer formation (C53, C63, C91)	Region needed for NFκβ activation (8–11)
ISG15	Site needed for disulphide bond (C78) and signalling (Y96, R99, T101, T102, T103); LRLGG motif (152–187)	
MOV10	Region needed for interaction with AGO2 and APOBEC3g (995–998); DEAG box (678–681)	
OAS2	Sites needed for enzyme activity (D408 + D410, Y421, D481, R544, K547)	Sites needed for enzyme activity (669–670)
Viperin	Sites needed for antiviral activity against Zika virus (K358), ubiquitination (K206), acetylation (K197) and protein stability (C83, C87, C90)	Leucine zipper motif (14–42)
TRIM22	Sites needed for antiviral activity (C15, C18)	Nuclear localisation signal (257–275), Site important for formation of nuclear bodies (V943)

### 2.3 Characterisation of Novel Ferret ISGs and ISTs

**FIGURE 4:**
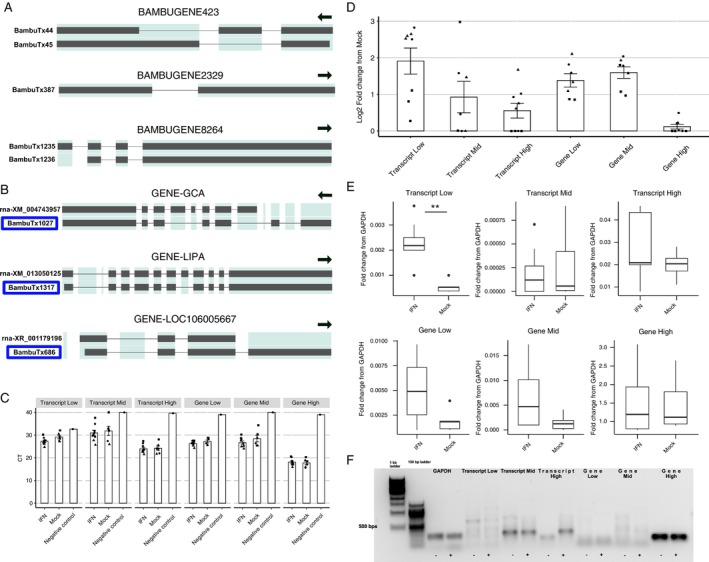
Novel ISGs and ISTs derived from *Bambu* analysis. (A) Novel ISGs in order of expression—*BambuGene423* (low), *BambuGene2329* (mid) and *BambuGene8264* (high). *BambuGene8264* revealed exceptionally high expression compared with the other novel ISGs with two main novel isoforms. Arrows indicate the direction of the strand from 5′ to 3′. (B) Novel ISTs in order of expression—GCA—*BambuTx1027* (low), *LIPA*—*BambuTx1317* (mid) and *LOC106005667*—*BambuTx686* (high). Novel transcripts from known genes are highlighted in blue boxes. Only reference transcripts with the highest expression are shown for comparison. Arrows indicate the direction of the strand from 5′ to 3′. (C) Raw CT values of novel ISTs and ISGs from RNA extracted from mock‐ or IFN‐treated FRL cells. Negative controls (primer only) have been included for comparison. (D) Log_2_ fold change in expression levels relative to mock controls, calculated using 2^−ΔΔCT^ method and (E) fold change relative to housekeeping gene *GAPDH* using the 2^−ΔCT^ method. Pooled data from three independent experiments are shown, different symbols are used to denote each experiment. Statistical differences in CT and Fold change value from mock (C and E) were calculated using a mixed effects model. *p < 0.05, **p < 0.01, ***p < 0.001. (F) Representative DNA gel to visualise qPCR products from in vitro samples.

In addition to visualising ferret orthologous of human ISGs, we also searched for novel unannotated ISGs and ISTs in the datasets generated. We focused our analyses on novel genes and transcripts (‘BambuGene’ and ‘BambuTx’) upregulated in the in vitro data set (IFN‐α stimulation, *p*_adj_ < 0.05), reasoning that these were most likely to represent potential ISGs and ISTs. Genes and transcripts of interest were (i) ranked via estimated average expression level in the IFN‐treated datasets and then (ii) selected for low, mid and high expression genes and transcripts depending on their rank position (Figure 4A,B). Through this process, we selected novel transcripts from three genes—*GCA*, *LIPA* and *LOC106005667*. The novel ISTs showed exon‐skipping events and alternative 5′ ends compared to the annotated transcripts (Figure 4B). The selected ISGs and ISTs were then validated using PCR and RT‐qPCR to confirm a trend towards higher expression in datasets from IFN‐treated compared to mock‐treated FRL cells for most ISGs/ISTs, although this was not significant (Figure 4C–E). Additionally, the novel gene *BambuGene8284* appeared to show remarkably high expression levels in datasets from both mock and IFN‐treated FRL cells (Figure 4F), which further validated the existence of the transcripts. Analysis of gene expression data from sequencing showed that this gene also was upregulated in the nasal turbinates of IAV‐infected ferrets and expression levels increased markedly between 6 and 24 hpi (Figure S2). Several other *BambuGenes* were also upregulated following IFN treatment (in vitro) or IAV infection (in vivo) (Figures S2 and S3) and future studies will determine their coding potential and their role in modulating IAV infection. Overall, our analysis reveals the presence of novel ISGs and ISTs that have not yet been annotated in the reference ferret genome and transcriptome.

### 2.4 Novel Trans‐Spliced Paralogous Reads in Potential Ferret Interferon‐Induced Transmembrane Protein ( *IFITM* ) Genes

**FIGURE 5:**
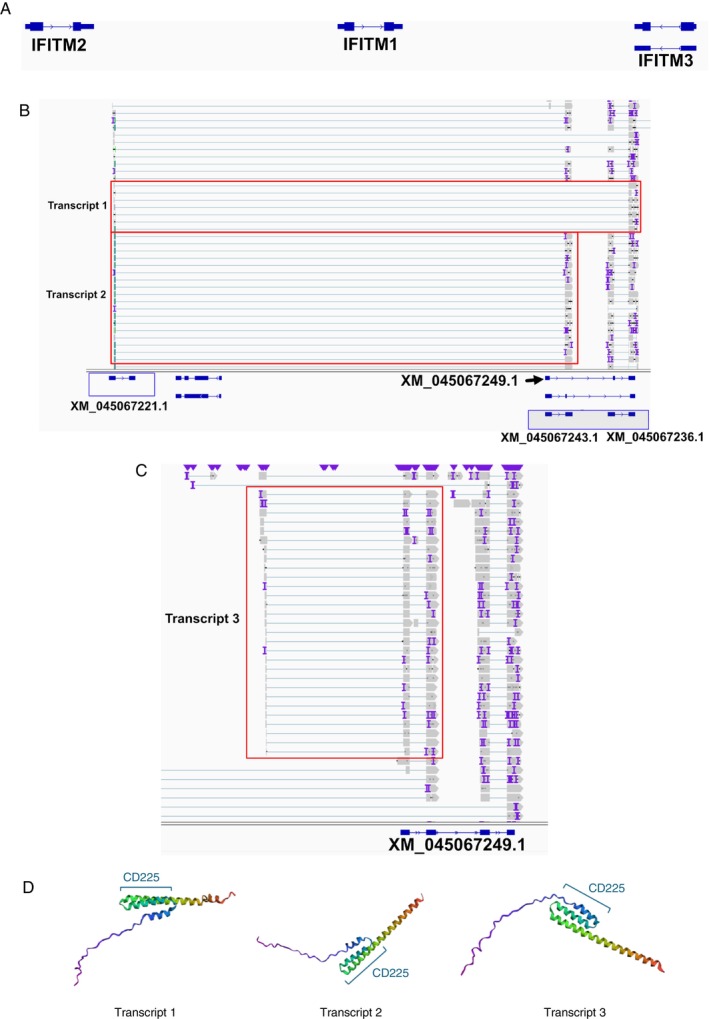
Potential novel trans‐spliced transcripts in the ferret *IFITM* family of genes. (A) *IGV* plot visualisation of human *IFITM1‐3* reference transcripts. (B) *IGV* plot visualisation of putatively trans‐spliced *IFITM* transcripts (Transcripts 1 and 2). The relevant reads are highlighted via a red box. Reference transcripts that appeared to be involved in the trans‐splicing events are highlighted via the blue boxes. (C) *IGV* plot visualisation of reads suggestive of extra 5′ exons in putative ferret *IFITM* transcripts (Transcript 3). (D) Protein structure estimates of trans‐spliced transcripts using *trRosetta*.

The *IFITM* gene family encodes proteins that mediate antiviral activity, including against IAV, by limiting the early stages of the virus replication cycle [27]. In humans, three IFITMs (IFITM1, 2 and 3) have been particularly well characterised and are known to localise to plasma (IFITM1) or endosomal membranes (IFITM2 and 3) where they can inhibit entry by viruses that enter cells by direct fusion (IFITM1) or by endocytosis (IFITM2 and 3) [28]. In contrast, ferret IFITM genes are generally not well characterised and the transcript sequences available are predicted from short‐read data in NCBI. A recent study used qPCR to demonstrate that the hypothetical (i.e., ‘XM’) ferret IFITM transcripts were upregulated in IFN‐treated and IAV‐infected FRL cells [29]. Herein, we also observed similar results in both in vitro and in vivo datasets (Figure 2A,B). Compared to the well‐characterised human transcriptome (Figure 5A), the annotated hypothetical transcripts in ferrets revealed greater variety in potential orthologous regions (Figure 5B). Furthermore, upon visualisation using the *Integrative Genomics Viewer (IGV)*, we noted reads that mapped to exons of different hypothetical (i.e., ‘XM’) transcripts of *IFITM* genes, suggesting trans‐splicing events (Figure 5B). We also observed evidence of reads that showed additional 5′ exons compared with the annotated exons of genes (Figure 5C), implying an incomplete annotation of the *IFITM* transcripts. To understand the functional consequences of trans‐spliced transcripts and transcripts with extra 5′ exons (denoted Transcript 1, Transcript 2 and Transcript 3 in this manuscript), we predicted their protein structures. The resulting predicted proteins all contained a conserved CD225 sequence that regulates vesicular membrane fusion, contributing to the most important functional domain for IFITM, as also observed in the reference *IFITM* transcripts (Figure 5D) [30]. The major sequential differences between the three predicted proteins appeared to be in the N‐terminal region.

**FIGURE 6:**
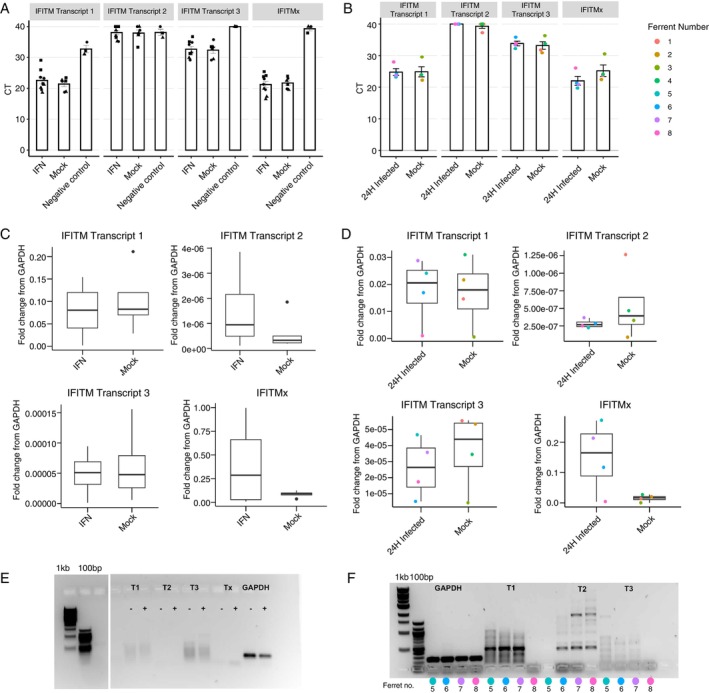
Validating the expression of novel trans‐spliced *IFITM* transcripts by RT‐qPCR. (A, B) Raw CT values of *IFITM* transcripts under different experimental conditions in FRL cells (A) or in ferret nasal turbinates (B). (C, D) Expression level of *IFITM* transcripts under different experimental conditions in FRL cells (C) or ferret nasal turbinates (D), with fold change relative to housekeeping gene *GAPDH* calculated using the 2^−ΔCT^ method. For in vitro data, pooled data from three independent experiments are shown (A/C) and different symbols are used to denote each experiment. For in vivo data, different colours indicate individual animals. Statistical differences in CT and fold change values from mock was calculated using a mixed effects model. *p < 0.05, **p < 0.01, *** *p* < 0.001. (E, F) Representative DNA gels to visualise qPCR products from in vitro (E) or in vivo (F) samples.

To investigate whether trans‐spliced *IFITM* transcripts were actually expressed in vitro and/or in vivo, we first designed primers specific to each novel trans‐spliced transcript (taking care to ensure the primers were specific; Figure S4). We then utilised RT‐qPCR and RNA extracted from mock‐ or IFN‐treated FRL cells or mock‐ or IAV‐infected nasal turbinates of ferrets to determine expression levels (Figure 6). In our in vitro dataset, we noted that a transcript sequence in NCBI labelled as ferret *IFITM3* (XM_045067236.1) was highly upregulated in FRL cells following IFN treatment. However, as this transcript exhibits a high degree of sequence similarity to both human *IFITM2* and *IFITM3*, we subsequently refer to this as *IFITMx* in our studies. Due to its high expression levels, we utilised *IFITMx* as a positive control in the RT‐qPCR assays below. Compared to negative controls, RT‐qPCR results from FRL cells demonstrated an appreciable difference in CT values for *IFITM* Transcript 1, but not Transcripts 2 and 3, suggesting they are not being expressed at levels detectable by RT‐qPCR (Figure 6A). In contrast, *IFITMx* was readily detected by RT‐qPCR. Interestingly, none of the ferret *IFITMs* were expressed at high levels relative to the housekeeping gene *GAPDH* (Figure 6C), even following IFN stimulation of FRL cells, although it should be noted that *IFITMx* expression did increase (~2‐fold relative to mock) in response to IFN treatment (Figure S5). Visualisation of the RT‐qPCR products showed a distinct band for *GAPDH* and a faint, but distinct, band for *IFITMx* which increased in intensity following IFN treatment. However, no clear bands could be observed for any of the novel *IFITM* transcripts (Figure 6E). The trends in CT and fold change derived from in vivo samples were similar to those from in vitro samples (Figure 6B,D), with *IFITMx* showing a ~5‐fold increase relative to mock in IAV‐infected samples, noting that this was not significant (Figure S5). Visualisation of RT‐qPCR products showed a distinct band for *GAPDH* and *IFITM* Transcript 1, but not for *IFITM* Transcript 2 or 3 (Figure 6F). Together, these results suggest that *IFITM* Transcript 1 is produced, with stronger evidence that this trans‐spliced transcript is produced in vivo, although it does not appear to be upregulated by IFN treatment (in vitro) or IAV infection (in vivo).

### 2.5 Ferret Poly(A) Tail Regulation Is Associated With Viral Infection Pathways

**FIGURE 7:**
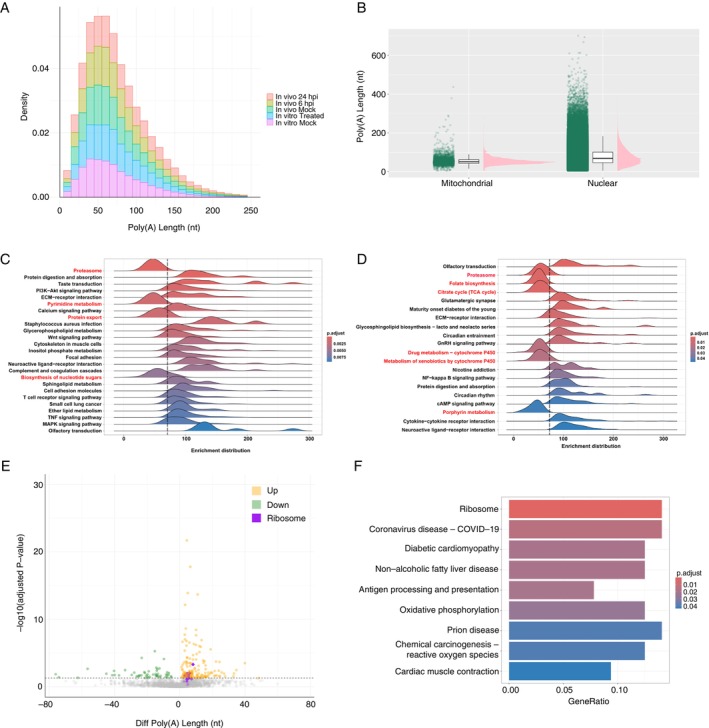
Polyadenylation states in the ferret transcriptome. (A) Density distributions of poly(A) lengths in each dataset. Only nuclear RNA has been included. (B) Poly(A) distributions between nuclear and mitochondrial RNA. A random sample of 300 000 reads across the different datasets were used. (C, D) KEGG pathway GSEA results using (C) in vitro mock‐control and (D) in vivo mock‐control data. Pathways highlighted in red are those in which the maximum density peaks are deemed with lower average poly(A) length (indicated by the dashed vertical line). The genes were ranked based on poly(A) lengths. (E) Differential polyadenylation results between in vitro 24 hpi IFN‐treated versus mock‐control data. *x‐*axis shows the difference in maxpeak poly(A) length between the two datasets compared and the *y*‐axis shows the ‐log_10_ padj, with a threshold of *p*_adj_ < 0.05. Each dot represents a gene, where green = decreased polyadenylation, yellow = increased polyadenylation and purple = genes involved in the *ribosome* KEGG pathway, shown to be increased in polyadenylation. (F) KEGG pathway enrichment analysis of genes with increased poly(A) tail lengths upon IFN‐treatment compared with mock‐control in the in vitro dataset with a threshold of *p*_adj_ < 0.05.

A favourable feature of ONT RNA‐seq is the ability to estimate transcriptome‐wide poly(A) tail length. As such, we investigated the lengths of the poly(A) tails of ferret mRNA. All datasets, despite having varied conditions, showed comparable distributions (Figure 7A). The ferret transcriptome also had a median of ~52 nt in the mitochondrial genes and ~68 nt in the nuclear genes, which agrees with studies in human polyadenylomes [14, 16, 17] (Figure 7B). The in vivo dataset (IAV‐infected ferrets at 24 hpi) also exhibited the highest proportion of reads with poly(A) tails greater than 400 nt (Table 2), which suggests a correlation between infection and poly(A) tail elongation, in line with previous work in human cell lines [14]. To determine if the poly(A) tail length varied depending on Kyoto Encyclopedia of Genes and Genomes (KEGG) pathways, we used the Gene Set Enrichment analysis tool to identify pathways enriched for shorter or longer tails in mock/control conditions only. Interestingly, two main groupings were apparent based on the maximum peak in density of poly(A) tails; a smaller subset of pathways with shorter maximum densities below ~70 nt and a larger group of pathways with longer maximum densities (Figure 7C,D). This indicates that as in other species, the ferret polyadenylome appeared to have a distinct set of genes which harbour shorter or longer poly(A) tails depending on their functions. Notably, both mock‐treated FRL cells (Figure 7C) and mock‐infected ferret nasal turbinates (Figure 7D) showed the *proteasome* pathway as being part of the shorter length poly(A) group and other metabolic pathways. Additionally, differential polyadenylation analysis revealed that IFN‐treated FRL cells had elongation of poly(A) tails in the mRNA from genes involved in the *ribosome*, *coronavirus disease 19* and *oxidative phosphorylation* KEGG pathways, in comparison to mock‐treated FRL cells, revealing a potential relationship between poly(A) tail elongation and innate immune responses in domestic ferrets (Figure 7E,F). Overall, the polyadenylome of ferrets aligns with the human polyadenylome [17], showing its feasibility as a model organism and as a proxy for understanding human responses to IFN treatment or IAV infection.

**TABLE 2:** Number of reads belonging to each dataset and reads with long poly(A) tails (> 400 nt).

Dataset	Total read count	#Reads with poly(A) > 400 nt	Proportion of reads with poly(A) > 400 nt
In vitro‐mock	5 309 690	729	1.37E−04
In vitro‐IFN treated	5 026 980	562	1.12E−04
In vivo‐mock	4 982 784	1095	2.20E−04
In vivo‐IAV infection, 24 hpi	11 395 103	4270	3.74E−04
In vivo‐IAV infection, 6 hpi	12 330 668	3120	2.53E−04

## 3 Discussion

The European domestic ferret ( *M. p. furo* ) reference genome and transcriptome have been curated predominantly through computational predictions, despite the ferret being a benchmark species for IAV infections [24]. This was clearly apparent due to the proportion of known versus hypothetical transcripts detected via our *Bambu* analysis, which identified known (‘NM’: 64, ‘NR’: 0) and hypothetical (‘XM’: 57 475, ‘XR’: 10 942) transcripts. This makes investigating ferret responses to different stimuli using multi‐omic approaches very challenging. In this study, we comprehensively evaluated the ferret transcriptome using ONT long‐read RNA sequencing, which validated the annotated ferret transcripts in NCBI, as well as detecting novel transcripts through our reference‐guided analyses. We detected over 1400 novel transcripts, confirming the importance of utilising long‐read technology to assess an incomplete transcriptome. We also observed increased diversity in transcripts detected in the in vivo data (Figures 1C and S1B,D), which may be caused by the greater variation between individual ferrets compared with the homogenous FRL cell line. Moreover, the nasal epithelia is composed of heterogeneous cells, including ciliated, secretory and basal cells [31], as well as a varied composition of immune cells both in homeostasis and during infection, contributing to the increased diversity. We note that assessing the bulk response of these cell populations by averaging across cell types will mask the intricacies of cell‐type‐dependent transcriptional regulation. Therefore, further research should be conducted to assess the IST expression at a single‐cell level in the ferret nasal epithelia.

Previous studies investigating ferret ISGs in the context of viral infections have been hampered by two main challenges: (i) the inability to determine which ferret ISGs are genuinely expressed during viral infection and (ii) the existence of multiple predicted transcript variants for each ISG in the NCBI database, with no indication of which isoform represents the major transcript produced during infection or in response to IFN stimulation [8, 29]. For example, in our previous study on ferret Mx proteins, we were unable to characterise ferret *Mx2* at the protein level due to the presence of 14 predicted transcript variants on NCBI, making it difficult to identify the biologically relevant isoform for functional studies. Similarly, a prior study aiming to characterise ferret *IFITMs* first required annotation of the transcript sequences from the gene locus before RT‐qPCR validation of their expression could be performed [29]. With these limitations in mind, the first aim of this study was to identify the major transcript variants of ferret ISGs that are orthologous to human ISGs with known anti‐IAV activity. We successfully identified the dominant transcript variants of ferret *Mx1* (previously characterised in [8]), *Tetherin, ISG15, MOV10, TRIM22, Viperin* and *OAS2*. Using RT‐qPCR, we demonstrated that all of these ferret ISGs, except *MOV10*, were upregulated following IFN treatment. Unfortunately, we were not able to determine the major transcript variant of ferret *Mx2*, due to this transcript being very long and poor sequencing coverage in the N‐terminal region where most variation between the transcript variants is observed.

Of note, the initial ferret *OAS* transcript identified (XM_004753413.2, annotated as *OAS3* on NCBI) in this analysis appeared unusually long and resembled a fusion between human *OAS2* and *OAS3*. Upon further investigation, we determined that there was stronger evidence of a shorter transcript being present, corresponding to region 3456‐6750 of XM_004753413.2. This shorter transcript showed a high sequence similarity to human *OAS2*. Accordingly, subsequent RT‐qPCR analysis and protein alignment studies focused on this transcript, which we refer to as ferret *OAS2* in this manuscript. Notably, there is currently no ferret *OAS2* annotated in the NCBI database, making this a novel finding. Similarly, we identified a ferret *IFITM* sequence (XM_045067236.1) that has not been described in the previously published Horman et al. paper [29] and has a sequence similar to both human *IFITM2* and *IFITM3*. We have denoted this as *IFITMx* in this manuscript. In addition, we have some evidence to indicate that trans‐spliced *IFITM* transcripts are produced in ferrets, which were most prevalent in IAV‐infected ferrets and were not strongly induced by IFN treatment in FRL cells. While protein structural analysis suggests that these trans‐spliced transcripts can encode functional proteins, whether they play a role in virus infections remains to be determined. Trans‐splicing events can lead to novel protein formation, creating neoantigens that may elicit a varied or greater immune response. Otherwise, the trans‐spliced transcripts can act as regulatory non‐coding RNA and participate in increasing the genome complexity. Taken together, while these transcripts were rather poorly expressed in comparison with reference *IFITM* transcripts, we hypothesise that the trans‐spliced *IFITM* transcripts may act as backup transcripts to encumber diversity within *IFITMs*, to act against IAV infections.

In addition to identifying the dominant transcript variants of ferret ISGs, we were able to compare the protein coding region of the ferret ISGs with other mammalian species, such as humans, mice and European mink. As expected, there was a high degree of similarity between ferret and mink ISGs, noting that the mink database is also incomplete. This incompleteness likely played a role in the high degree of divergence observed between mink and ferret *OAS2*. Regardless, the information gleaned from this study and future studies characterising the antiviral activity of ferret ISGs in more detail is likely to provide additional insights into mink ISGs as well.

In addition to comparing protein coding sequences, our alignment data also provided us with some potential insights into differences in protein function and/or localisation between ferret and human ISGs. For example, data from Table 1 of this study indicate that ferret *TRIM22* contains mutations within the nuclear localisation signal present in human *TRIM22*. As a result, unlike its human counterpart, ferret *TRIM22* may not localise to the nucleus during infection [32]. Further experiments are needed to confirm this hypothesis and determine if ferret *TRIM22* has similar antiviral activity to human *TRIM22* or not.

In addition to identifying orthologues of known ISGs, we were able to find unannotated ISGs and ISTs which were upregulated following IFN treatment of FRL cells and/or IAV infection of ferrets (Figures S2 and S3), with *BambuGene8284* showing impressively high baseline expression levels in vitro and in vivo. While this novel gene does not align with many of the known ISGs in humans, *Blastn* analysis suggests that this gene encodes a noncoding RNA, which is of interest as ncRNAs are increasingly recognised as ISGs with the potential to modulate viral infection [33]. Further work will be required to fully elucidate the characteristics of these novel ISGs and ISTs.

Polyadenylation is an important co‐transcriptional modification which occurs not only in mammals but also in viruses [10, 34] and plants [35]. According to our previous studies on the polyadenylome of the human transcriptome [14, 17], we found a correlation between the ferret polyadenylome and the human polyadenylome in terms of general distribution and effect during an event such as IAV infection and/or IFN treatment. Particularly, IFN‐treated FRL cells exhibited increased poly(A) tail lengths of mRNA of genes related to KEGG pathways commonly associated with viral or bacterial infection, such as *ribosome*, *COVID‐19* and *oxidative phosphorylation* (Figure 7E,F). This is in alignment with our previous results with Calu‐3 cells infected with SARS‐CoV‐2 [14]. Poly(A) tail length has been correlated with the stability of the mRNA transcript [22] and suggests that this mechanism is part of the anti‐viral arsenal of the host or contributes to the viral manipulation of host ribosome functions. However, no significant pathway enrichment was found in the in vivo datasets. This suggests (i) a host‐specific activity of the polyadenylome, (ii) the species‐dependent response to viral infection or (iii) differences in responses to viral infection vs. IFN treatment. Extending this work to determine the transcriptomic changes in IAV infection vs. IFN treatment in both in vitro and in vivo settings would be beneficial to understand these differences, in addition to understanding the aforementioned expression‐level differences.

To date, only two studies have examined the ferret transcriptome following viral infection. One study sequenced and annotated the ferret transcriptome in lung lobes and lymph nodes after IAV infection [23], while another investigated transcriptomic responses in ferret lungs following henipavirus infection [36]. Of note a third study measured innate immune responses in ferret lung following enterovirus infection, but used qPCR analysis instead of transcriptomics [37]. The earlier study by León et al. was, particularly, instrumental in developing the ferret genome, with many current NCBI transcript annotations derived from that work. However, both studies used Illumina short‐read sequencing technology, which limits the ability to resolve full‐length transcripts and detect transcript isoforms. Furthermore, both focused exclusively on lung tissue, despite the nasal epithelium being a key site of IAV replication and a site whose transcriptomic response remains largely unexplored.

To address these gaps, we conducted long‐read sequencing on ferret lung cells ± IFN treatment (in vitro), as well as ferret nasal turbinates ± IAV infection (in vivo). While a recent study [38] used long‐read sequencing to characterise the ferret immune repertoire, focusing on IgG and T cell receptor transcripts in splenocytes and lymph nodes, our study is, to our knowledge, the first to apply this technology to profile early innate immune responses in ferret nasal tissues. A limitation of our study was the lower RNA integrity (RINe) scores in the nasal turbinates samples, due to the sample collection method, which likely contributed to shorter N50 values. Nonetheless, we achieved sufficient coverage to capture full‐length transcripts. Furthermore, due to the PCR amplification of our transcripts, only relative quantification was able to be carried out due to potential PCR bias. While unique molecular identifiers (UMIs) were incorporated into the protocol to collapse the PCR duplicates, the UMI detection rate was insufficient to perform the deduplication, which may be caused by the higher error rates of the early version of the PCR cDNA barcoding kit. Despite this, amplified data is beneficial for detecting novel transcripts, which was the key mission of this study. This work may be extended by utilising the most recent version of the ONT kit or direct RNA‐sequencing, leading to higher accuracy and an enhanced view of the ferret transcriptome.

Finally, while transcriptomic profiling provides a comprehensive view of gene expression dynamics, including the identification of novel and orthologous ISGs, it does not always correlate directly with protein abundance or activity. This is, particularly, relevant in the context of ferrets, where inter‐individual variation and post‐transcriptional regulation may influence the functional outcomes of infection. However, proteomic analysis in ferrets remains technically challenging due to limited species‐specific reagents and annotated protein databases. Accordingly, we employed long‐read transcriptomics to maximise isoform resolution and gene discovery, which is a critical first step in characterising the ferret immune response. Future work integrating proteomic approaches, such as mass spectrometry or over‐expression assays, will be essential to validate the functional relevance of these ISGs and to further establish the ferret as a translational model.

Overall, our study represents the first long‐read transcriptomic profiling of early antiviral responses in ferret respiratory tissues and provides an essential resource for improving gene annotation, understanding ISG expression dynamics and supporting future functional studies in this important animal model.

## 4 Methods

### 4.1 Cells and Viruses

FRL cells, an adenovirus 5‐immortalised cell line (kindly provided by Prof. Tuck‐Weng Kok, University of Adelaide), were maintained and passaged in Dulbecco's modified Eagle medium (DMEM) (Gibco) supplemented with 10% (v/v) FBS, 2 mM L‐glutamine (Gibco) and 1 mM sodium pyruvate (Gibco). Madin‐Darby Canine kidney (MDCK) cells (ATCC CCL‐34) were maintained and passaged in RPMI 1640 medium supplemented with 10% (v/v) foetal bovine serum (FBS, Gibco), 2 mM l‐glutamine and 1 mM sodium pyruvate.

The seasonal IAV strain used in this study A/Perth/265/2009 (Perth/09, H1N1pdm09) was obtained from the WHO Collaborating Centre for Reference and Research on Influenza (WHO CCRRI), Melbourne, Australia. Viruses were propagated in the allantoic cavity of 11‐day embryonated chicken eggs following standard procedures [24] and titres of infectious virus were determined on MDCK cells by standard plaque assay and expressed as plaque‐forming units (PFU) per mL [31].

### 4.2 Detection of Ferret ISGs by qPCR Following In Vitro Induction

To test for induction of ferret ISGs in vitro, FRL cells were seeded in six‐well plates 24 h before treatment with 100 ng/μL of recombinant ferret IFN‐α (a kind gift from Tim Adams, CSIRO Manufacturing, Parkville, Australia). At 24 h post‐treatment, total RNA was extracted using the RNeasy Plus Mini Kit (Qiagen) and treated with amplification‐grade Dnase I (Sigma Aldrich) and RNA concentration was then standardised across samples. RNA was then converted to cDNA using the SensiFAST cDNA Synthesis Kit (Bioline). SYBR green‐based qPCR was used to determine the expression of ferret genes relative to the housekeeping gene *GAPDH* (glyceraldehyde 3‐phosphate dehydrogenase) using the SensiFAST SYBR kit (Bioline). Data acquisition was performed using the QuantStudio 7 Flex Real‐Time PCR System (Applied Biosystems). For visualisation, qPCR products were run on a 0.8% agarose gel. Primer sequences used for qPCR are as below:

**Table:** 

Gene spanning primers		Forward	Reverse
IFITM_potential_transcript_1		GGCCACTGCCCACAGCC	TTAACCAACTGAGCCACCC
IFITM_potential_transcript_2		CGACGGAGACGCCTGTGCGG	CCAGAAAAACAACAGATACAG
IFITM_potential_transcript_3		AAAACCCAAGGCCCCAAGAG	CCGAGTGGGTGAGTCTGATG
Novel genes	Expression	Forward	Reverse
Bambu423	Low	TTGAGGCCGGAGTTCAATGA	GGGGATTTATTCATTTCCGGGC
Bambu2329	Med	ACAACCACCGAGGATGCAAA	GGGCAGCCCTTATTGCTAGA
Bambu8264	High	CCAGGGACGCTGTTACCAAT	CCCGAAAAGTCACAGGTCCA
Novel Transcript	Expression	Forward	Reverse
BambuTx686	High	TACAGAGGGCTGCAAAGCAA	TTGAATATGCAGCACTGTGGC
BambuTx1317	Med	CACAGTGGCCCGGGAGGACC	AAACAAGTCCTTCAAGAGAAATTC
BambuTx1027	Low	TTCGGTCGGCCGTGGC	GCATCACGCGCTTCCAAGC
ISG		Forward	Reverse
Ferret_Tetherin		TCCTAGCAGAGCAGGAGTGT	CTCCTCTCTTCCCCTGAGCT
Ferret_ISG15		ATAGCCCAGAAAACTGGCGT	ACCCTTGTCGTTTCTCACCA
Ferret_MOV10		CGTCATCCTCATCTCCACCG	TTTCCAGTCAGGGTCATGGC
Ferret_OAS2		GAGCCCTGGACATGACCAAA	GGGACAGACCTTTCAGCCTC
Ferret_Viperin		AGTCAATGTCCTCATCGGCC	CACTTTCCAGCGGACAGGAT
Ferret_TRIM22		ACCTGTTCCTCCCTGTCGTA	CTCGGTGGGCACAAGGTTAT
Ferret_IFITMx		GGGCCTCCTGCTGACCAT	CGGAATCATCAGCGATCCA

### 4.3 Ethics Statement

Ferret experiments were conducted with approval from the University of Melbourne Biochemistry and Molecular Biology, Dental Science, Medicine, Microbiology and Immunology and Surgery Animal Ethics Committee (AEC# 20033), in accordance with the NHMRC Australian code of practice for the care and use of animals for scientific purposes.

#### 4.3.1 IAV Infection of Ferrets

Adult outbred ferrets (600–1500 g) were housed in the Bioresources Facility at the Peter Doherty Institute for Infection and Immunity, Melbourne, Australia. Before the commencement of experiments, hemagglutination inhibition assays were used to confirm all animals to be seronegative against IAV strain A/Perth/265/2009 (Perth/09, H1N1)pdm09. For IAV infection, 12 ferrets were anaesthetised (25 mg/kg ketamine and 5 mg/kg ilium Xylazilin in a 1:1 (vol/vol) mixture) and 8 were inoculated by dropwise intranasal delivery of 500 μL of PBS containing 10^7^ PFU of Perth/09 and 4 were mock‐infected with an equivalent volume of PBS. At either 6 or 24 h post‐infection, ferrets were euthanised for collection of nasal tissues. For euthanasia, ferrets were anaesthetised using a mixture of ketamine and xylazine following pentobarbitone sodium (Lethabarb Troy Laboratories) injection. Sections of nasal tissues were stored in 5 mL RNALater overnight at 4°C and tissues were then frozen at −80°C. Total RNA was extracted from ferret nasal tissue samples using the RNeasy Mini Plus kit (Qiagen) according to the manufacturer's instructions. Briefly, 5 or 3 mL RLT buffer containing 143 mM β‐mercaptoethanol was added directly to the nasal tissue samples in gentleMACS M tubes (Miltenyi Biotec). Samples were homogenised using the gentleMACS dissociator (Miltenyi Biotec) and lysates were clarified twice by centrifugation at 3000*g* for 10 min. RNA was extracted using the animal tissue protocol and eluted in 50 μL. RNA concentration and purity were assessed by spectrophotometry (*A*_260_/*A*_280_).

#### 4.3.2 RNA Extraction and DNAse‐Treatment for ONT

Total RNA was extracted from cellular material using the RNeasy Mini Kit (Qiagen), following the manufacturer's guidelines. The extracted RNA was treated with DNase using the Turbo DNA‐free kit (Ambion). QC was carried out using the Qubit RNA High Sensitivity Assay (ThermoFisher Scientific) and Qubit 1 × dsDNA High Sensitivity Assay (ThermoFisher Scientific) on the Qubit 4 Fluorometer (ThermoFisher Scientific), High Sensitivity RNA and High Sensitivity D5000 Assays on the Tapestation 4200 System (Agilent Technologies) and NanoDrop 2000/2000 Spectrophotometer (ThermoFisher Scientific).

#### 4.3.3 ONT Sequencing

DNAse‐treated RNA was used as input for the PCR‐cDNA Barcoding Kit (SQK‐PCB111.24, ONT) according to the manufacturer's guidelines, with minor modifications. Modifications include the increased input RNA amount to 500 ng instead of the 200 ng in the protocol, as well as using 18 cycles and 10 min extension times for the PCR step. Additionally, synthetic RNA (Sequin MixA) was added at 5% of the expected level of mRNA (5% total RNA) [39]. PCR artefacts were removed using the prescribed method from ONT. The sequencing was carried out with R9.4.1 MinION flow cells on the ONT GridION for 72 h.

## 5 Data Analysis

### 5.1 Basecalling

Original passing threshold fast5 (*Q*_score_ ≥ 10) output from the GridION was converted to pod5 format using pod5 tool to boost the efficiency of basecalling. *Dorado* (v0.5.3 or v0.9.6) was used to perform basecalling on the pod5 signals with the output format as ubam for quantification and poly(A) tail length estimation, respectively. ‘dna_r9.4.1_e8_sup@v3.6’ was the selected model and ‘‐‐estimate‐poly‐a’ parameter was raised to estimate poly(A) tail length for each read in the sequence. ‘samtools (v1.16.1) fastq’ command converted the ubam to fastq with ‘‐T *’ parameter to keep all the tag information [40].

### 5.2 Restranding and Reference Mapping

Before reference mapping, *Restrander* (v1.0.1) [41] helped to remove artefact reads in our ONT long‐read cDNA sequences, using the prebuild configuration for the PCB111 protocol. Reference alignment was performed by *minimap2* (v2.24) [42] with parameters as ‘‐y ‐t 20 ‐‐eqx ‐k 12 ‐uf ‐ax splice ‐‐secondary=no’. The ferret reference genome used was downloaded from NCBI (Genome assembly ASM1176430v1.1, taxon as *M. p. furo* (domestic ferret)). To keep the primary mapping reads only, ‘samtools view ‐F 2308’ was run to filter for the mapped reads.

### 5.3 Quantification and Isoform Discovery

*Bambu* (v3.2.4) package in R was performed for multi‐sample transcript discovery and quantification [43]. Gene and transcript expressions were calculated for each sample. The extended annotation output from *Bambu* was used as input for *SQANTI3* (v5.1.2) [44]. *Apptainer* (v1.2.3) was used to drive the *SQANTI3* container [45]. ‘sqanti3_qc.py’ and ‘sqanti3_filter ml’ were executed step by step to first classify all isoforms and then use a machine learning filter to remove artefacts. Then, the ‘rescue’ function was utilised to recover some transcripts in the artefact group. The quantification was carried out again using *Bambu* to generate the final count matrices and QC was carried out using *SQANTI3*.

*SQANTI3* isoform categories include Full Splice Match (FSM), Incomplete Splice Match (ISM), Novel In Catalogue (NIC), Novel Not In Catalogue (NNIC), Antisense, Genic Intron, Genic Genomic and Intergenic.

Each of these classes can be broadly defined as below:

- FSM—all splice junctions match perfectly to the reference transcript.
- ISM—the splice junctions match partially to the reference transcript.
- NIC—novel isoform formed from a combination of known splice sites.
- NNC—novel isoform with at least a new splicing site.
- Genic intron—transcript present within an intron.
- Genic genomic—transcript with overlap in intronic and exonic regions.
- Intergenic—transcript present in an intergenic region.

Novel genes and transcripts were visualised using *IGV* (v2.10.1) [46] and *IsoVis* [47]. The novel ISGs and ISTs which were selected for further validation via PCR were determined by isolating a list of BambuGenes and BambuTxs which were significantly upregulated in the in vitro comparison between the IFN‐treated versus mock‐control groups (*p*_adj_ < 0.05, see ‘differential expression and polyadenylation analyses’). Then, the mean expression level based on the transcript counts determined by *Bambu* between sequencing replicates was defined and the ISGs and ISTs were ranked based on this average statistic. For the ISGs, the low, mid and high genes were selected based on their ranking positioning. For the ISTs, the transcripts were selected based on whether the transcript was from an annotated gene and ranked based on the positions within this subset.

### 5.4 PolyA Tail Length Estimation

The estimated poly(A) tail lengths of each read were stored in the ubam files from the basecalling step. A combination of ‘samtools view’ and bash commands was implemented to extract the poly(A) tail lengths and sequence lengths into a text format via ‘samtools view my.bam | awk ‘/pt:i/{print $1,length($10),$NF‐1}’ | sed ‘s/pt:i://g’.

### 5.5 Differential Expression and Polyadenylation Analyses

Counts for duplicate transcripts were first merged in the counts matrices for transcripts. *DEseq2* (v1.38.3) [48] package in *R* (v4.2.1) was utilised to test differential expression between different conditions, where a *p*_adj_ threshold of 0.05 was used. *ggplot*, *pheatmap* and *ComplexHeatmap* packages in *R* were used to produce the volcano plots and heatmap plots.

The differential polyadenylation analysis aims to identify variants in poly(A) tail lengths across different sample conditions. By comparing two sets of paired conditions in both in vivo and in vitro samples, the analysis elucidated changes in polyadenylation patterns. From the basecalled data, *IsoQuant* (v3.3.1) [49] was employed to assign cDNA reads to genes. After cross‐matching poly(A) tail length information and gene assignment, we selected genes with more than 100 reads assigned and at least 10 reads assigned to a single sequencing replicate included in the comparison. The *R* package *lmerTest* (v3.1.3) [50] conducted a linear mixed‐effects regression (lmer), where the log‐transformed poly(A) length for all reads mapped to one gene served as the response variable, while the type of infection or treatment was the fixed effects and sample batch was the random effects. The per‐gene *p*‐values were generated and adjusted using the Benjamini–Hochberg (BH) method with the ‘p.adjust’ function in *R*. The differences between the mean of poly(A) tail length from each condition on each gene were calculated using *R*. Volcano plots were constructed using the *R* package *ggplot2* (v3.5.1) by setting the p.adjust value cut off ≤ 0.05 as significant.

### 5.6 Gene Set Enrichment and KEGG Pathway Enrichment Analyses

GSEA analysis for polyadenylation was implemented with the *ClusterProfiler* package in *R* [51]. The input data was the lmerTest gene list ordered by the difference of the average poly(A) tail for a condition (‘diff’) for each comparison in the differential polyadenylation analysis. The KEGG database for the *M. p. furo* (domestic ferret) (https://www.kegg.jp/kegg‐bin/show_organism?menu_type=pathway_maps&org=mpuf) was implemented for the enrichment analysis. For the KEGG pathway analysis, significant genes discovered in the differential polyadenylation analyses were queried in the enrichment analysis with background gene lists containing only genes that were involved in the differential analysis. Significant genes were separated into subgroups by upregulation and downregulation in different comparison groups.

### 5.7 Protein Structure Analysis

The sequences of hypothetical trans‐spliced transcripts were analysed for potential open reading frames (ORF) via the NCBI *ORFfinder* tool. The most robust protein sequences based on existing reference *IFITM* transcripts were selected for protein structure predictions via the *trRosetta* tool [52].

### 5.8 RT‐qPCR Data Analysis and Gene Alignments

Graphs and statistical analysis (as indicated in the figure legends) were generated using R‐Studio. Statistical analysis was performed by utilising a mixed effects model (*nlme* package in *R*). Protein sequence alignments were performed using the *MUSCLE* alignment function on *Geneious Prime* (v2025.0.2). The sequences used for the protein alignment are as follows (of note, the ORF sequence from the ferret transcripts was first translated in silico):

**Table:** 

Gene	Ferret	European mink	Humans	Mouse
BST2	XM_045081929.1	XP_059016426.1	NP_004326.1	NP_932763.1
ISG15	XM_004781282.3	XP_058994206.1	NP_005092.1	NP_056598.2
Viperin	XM_004745742.3	XP_058988730.1	Isoform 1: NP_542388.2, Isoform 2: NP_001397631.1	NP_067359.2
MOV10	Isoform 1: XM_045081891.1 Isoform 2: XM_004769794.3	XP_058993685.1	NP_001123551.1	NP_032645.2
OAS2	XM_004753413.2, region‐3456‐6750	XP_058995097.1	Isoform 1: NP_058197.2, Isoform 2: NP_002526.2	Isoform 1: NP_660262.2, Isoform 2: NP_001334377.1
TRIM22	XM_004778994.3	XP_059038208.1	Isoform 1: NP_006065.2, Isoform 2: NP_001186502.1	N/A

## Author Contributions

**Rubaiyea Farrukee:** conceptualisation, investigation, wet‐lab, writing. **Jessie J.‐Y. Chang:** conceptualisation, wet‐lab, analysis, writing. **Jianshu Zhang:** analysis, software, writing. **James B. Barnes:** wet‐lab, investigation. **Shu Xin Zhang:** wet‐lab. **Sher Maine Tan:** wet‐lab. **Patrick C. Reading:** conceptualisation, writing. **Lachlan J. M. Coin:** conceptualisation, writing.

## Conflicts of Interest

The authors declare no conflicts of interest.

## Supporting information


**Data S1:** imm70042‐sup‐0002‐Supinfo.txt.

**Figure S1:** Upregulated human ISG/HRF‐orthologous genes and transcripts in ferrets comparing IFN‐α‐treated vs mock‐control ferret FRL cells (in vitro) and nasal turbinate samples from IAV‐infected vs mock‐infected control ferrets (in vivo) at 6 and 24 hpi. (A) Differential expression results between 6 hpi IAV‐infected vs. mock‐infected control data (in vivo) at the gene level. (B–D) Differential transcript expression results between (B) IFN‐α‐treated versus mock‐control FRL cells (in vitro) and (C) 6 hpi (in vivo) and (D) 24 hpi IAV‐infected ferret versus mock‐infected control data (in vivo). *x* axis represents log_2_ fold changs and the *y* axis shows the −log_10_ *p*‐adjusted value (*p*_adj_), with a threshold of *p*_adj_ < 0.05, shown by the dotted horizontal line.
**Figure S2:** Heatmap of log_2_‐transformed gene expression values (CPM) across three in vivo conditions: mock‐treated, 6 and 24 h post‐infection (hpi). Each row represents a gene and columns represent the conditions. Expression patterns are annotated with two side colour bars representing logFC values at 6 and 24 hpi, respectively. Increase in purple intensity indicates higher logFC values at 24 hpi and increases in green intensity indicates higher logFC values at 6 hpi. Genes are ordered by decreasing logFC at 24 hpi. No row or column clustering was applied to preserve condition and ranking order.
**Figure S3:** Heatmap showing the log_2_‐transformed expression levels of selected genes in mock‐treated and IFN‐treated FRL cells from in vitro experiments. Expression values are based on CPM (counts per million) and are displayed for each gene across the two conditions. Genes are ordered by descending log fold change (logFC) in response to IFN treatment. The side colour bar represents logFC values, where green indicates strong upregulation following IFN treatment. Rows are not clustered to preserve the order of logFC.
**Figure S4:** Location of primer binding sites for novel trans‐spliced IFITM transcripts. (A) The forward (orange) and reverse (green) primers for Transcripts 1 and 3 bind to regions unique to the respective novel trans‐spliced transcripts. (B) For Transcript 2, although the individual forward (orange) and reverse (green) primers are not unique on their own, their specific combination produces a unique amplification product for Transcript 2.
**Figure S5:** Validating the expression of novel trans‐spliced *IFITM* transcripts by RT‐qPCR. Expression level of *IFITM* transcripts under different experimental conditions in FRL cells (A) or ferret nasal turbinates (B), showing log_2_ fold change in expression levels relative to mock controls, calculated using 2^−ΔΔ*C*T^ method. For in vitro data, pooled data from three independent experiments are shown (A) and different symbols are used to denote each experiment. For in vivo data, different colours indicate individual animals.

## Data Availability

All raw fastq sequencing datasets with poly(A) tail length tags are available on NCBI SRA at Project PRJNA1290003 and scripts are available on GitHub—https://github.com/abcdtree/ferret‐rna‐ont‐paper.

## References

[1] J. A. Belser , J. M. Katz , and T. M. Tumpey , “The Ferret as a Model Organism to Study Influenza A Virus Infection,” Disease Models & Mechanisms 4, no. 5 (2011): 575–579. 21810904 10.1242/dmm.007823 PMC3180220

[2] R. Farrukee , C. M. K. Tai , D. Y. Oh , et al., “Utilising Animal Models to Evaluate Oseltamivir Efficacy Against Influenza A and B Viruses With Reduced In Vitro Susceptibility,” PLoS Pathogens 16, no. 6 (2020): e1008592. 32555740 10.1371/journal.ppat.1008592 PMC7326275

[3] J. Skorupski , “Characterisation of the Complete Mitochondrial Genome of Critically Endangered *Mustela lutreola* (Carnivora: Mustelidae) and Its Phylogenetic and Conservation Implications,” Genes 13, no. 1 (2022): 125. 35052465 10.3390/genes13010125 PMC8774856

[4] M. Agüero , I. Monne , A. Sánchez , et al., “Highly Pathogenic Avian Influenza A(H5N1) Virus Infection in Farmed Minks, Spain, October 2022,” Eurosurveillance 28, no. 3 (2023): 2300001. 36695488 10.2807/1560-7917.ES.2023.28.3.2300001 PMC9853945

[5] H. Sun , F. Li , Q. Liu , et al., “Mink Is a Highly Susceptible Host Species to Circulating Human and Avian Influenza Viruses,” Emerging Microbes & Infections 10, no. 1 (2021): 472–480. 33657971 10.1080/22221751.2021.1899058 PMC7993395

[6] R. Farrukee , M. Ait‐Goughoulte , P. M. Saunders , S. L. Londrigan , and P. C. Reading , “Host Cell Restriction Factors of Paramyxoviruses and Pneumoviruses,” Viruses 12, no. 12 (2020): 1381. 33276587 10.3390/v12121381 PMC7761617

[7] F. Villalón‐Letelier , A. G. Brooks , P. M. Saunders , S. L. Londrigan , and P. C. Reading , “Host Cell Restriction Factors That Limit Influenza A Infection,” Viruses 9, no. 12 (2017): 376. 29215570 10.3390/v9120376 PMC5744151

[8] R. Farrukee , L. S. U. Schwab , J. B. Barnes , et al., “Induction and Antiviral Activity of Ferret Myxovirus Resistance (Mx) Protein 1 Against Influenza A Viruses,” Scientific Reports 14 (2024): 13524. 38866913 10.1038/s41598-024-63314-2 PMC11169552

[9] L. S. U. Schwab , S. L. Londrigan , A. G. Brooks , et al., “Induction of Interferon‐Stimulated Genes Correlates With Reduced Growth of Influenza A Virus in Lungs After RIG‐I Agonist Treatment of Ferrets,” Journal of Virology 96, no. 16 (2022): e0055922. 35916513 10.1128/jvi.00559-22 PMC9400473

[10] J. J. Chang , D. Rawlinson , M. E. Pitt , et al., “Transcriptional and Epi‐Transcriptional Dynamics of SARS‐CoV‐2 During Cellular Infection,” Cell Reports 35, no. 6 (2021): 109108. 33961822 10.1016/j.celrep.2021.109108 PMC8062406

[11] R. De Paoli‐Iseppi , S. Joshi , T. Wrzesinski , et al., “Using Long‐Read RNA Sequencing to Decipher the Role of RNA Isoforms in Disease,” Pathology 54 (2022): S17.

[12] J. Feng , W. Li , and T. Jiang , “Inference of Isoforms From Short Sequence Reads,” Journal of Computational Biology 18, no. 3 (2011): 305–321. 21385036 10.1089/cmb.2010.0243 PMC3123862

[13] Q. Lei , C. Li , Z. Zuo , C. Huang , H. Cheng , and R. Zhou , “Evolutionary Insights Into RNA Trans‐Splicing in Vertebrates,” Genome Biology and Evolution 8, no. 3 (2016): 562–577. 26966239 10.1093/gbe/evw025 PMC4824033

[14] J. J. Chang , J. Gleeson , D. Rawlinson , et al., “Long‐Read RNA Sequencing Identifies Polyadenylation Elongation and Differential Transcript Usage of Host Transcripts During SARS‐CoV‐2 In Vitro Infection,” Frontiers in Immunology 13 (2022): 832223. 35464437 10.3389/fimmu.2022.832223 PMC9019466

[15] J. J. Y. Chang , X. Yang , H. Teng , B. Reames , V. Corbin , and L. Coin , “Using Synthetic RNA to Benchmark Poly(A) Length Inference From Direct RNA Sequencing,” GigaScience 14 (2025): giaf098. 40899916 10.1093/gigascience/giaf098 PMC12406214

[16] H. Chang , J. Lim , M. Ha , and N. Kim , “TAIL‐Seq: Genome‐Wide Determination of Poly(A) Tail Length and 3′ End Modifications,” Molecular Cell 53, no. 6 (2014): 1044–1052. 24582499 10.1016/j.molcel.2014.02.007

[17] J. He , D. Ganesamoorthy , J. J. Chang , et al., “Utilizing Nanopore Direct RNA Sequencing of Blood From Patients With Sepsis for Discovery of Co‐ and Post‐Transcriptional Disease Biomarkers,” BMC Infectious Diseases 25, no. 1 (2025): 692. 40355874 10.1186/s12879-025-11078-z PMC12070577

[18] A. C. Beckel‐Mitchener , “Poly(A) Tail Length‐Dependent Stabilization of GAP‐43 mRNA by the RNA‐Binding Protein HuD,” Journal of Biological Chemistry 277, no. 31 (2002): 27996–28002. 12034726 10.1074/jbc.M201982200

[19] H. Fuke and M. Ohno , “Role of Poly (A) Tail as an Identity Element for mRNA Nuclear Export,” Nucleic Acids Research 36, no. 3 (2007): 1037–1049. 18096623 10.1093/nar/gkm1120 PMC2241894

[20] D. R. Gallie , “The Cap and Poly(A) Tail Function Synergistically to Regulate mRNA Translational Efficiency,” Genes & Development 5, no. 11 (1991): 2108–2116. 1682219 10.1101/gad.5.11.2108

[21] S. A. Lima , L. B. Chipman , A. L. Nicholson , et al., “Short Poly(A) Tails Are a Conserved Feature of Highly Expressed Genes,” Nature Structural & Molecular Biology 24, no. 12 (2017): 1057–1063. 10.1038/nsmb.3499 PMC5877826 29106412

[22] L. A. Passmore and J. Coller , “Roles of mRNA Poly(A) Tails in Regulation of Eukaryotic Gene Expression,” Nature Reviews. Molecular Cell Biology 23, no. 2 (2022): 93–106. 34594027 10.1038/s41580-021-00417-y PMC7614307

[23] A. J. León , D. Banner , L. Xu , et al., “Sequencing, Annotation, and Characterization of the Influenza Ferret Infectome,” Journal of Virology 87, no. 4 (2013): 1957–1966. 23236062 10.1128/JVI.02476-12 PMC3571481

[24] X. Peng , J. Alföldi , K. Gori , et al., “The Draft Genome Sequence of the Ferret ( *Mustela putorius furo* ) Facilitates Study of Human Respiratory Disease,” Nature Biotechnology 32, no. 12 (2014): 1250–1255. 10.1038/nbt.3079 PMC4262547 25402615

[25] S. S. Hussain , Y. J. K. Edwards , E. F. Libby , et al., “Comparative Transcriptomics in Human COPD Reveals Dysregulated Genes Uniquely Expressed in Ferrets,” Respiratory Research 23, no. 1 (2022): 277. 36217144 10.1186/s12931-022-02198-0 PMC9552453

[26] J. Wong , C. M. Tai , A. C. Hurt , H. X. Tan , S. J. Kent , and A. K. Wheatley , “Sequencing B Cell Receptors From Ferrets ( *Mustela putorius furo* ),” PLoS One 15, no. 5 (2020): e0233794. 32470013 10.1371/journal.pone.0233794 PMC7259655

[27] C. C. Bailey , G. Zhong , I. C. Huang , and M. Farzan , “IFITM‐Family Proteins: The Cell's First Line of Antiviral Defense,” Annual Review of Virology 1, no. 1 (2014): 261–283. 10.1146/annurev-virology-031413-085537 PMC4295558 25599080

[28] J. M. Perreira , C. R. Chin , E. M. Feeley , and A. L. Brass , “IFITMs Restrict the Replication of Multiple Pathogenic Viruses,” Journal of Molecular Biology 425, no. 24 (2013): 4937–4955. 24076421 10.1016/j.jmb.2013.09.024 PMC4121887

[29] W. S. J. Horman , K. Kedzierska , C. L. Rootes , A. G. D. Bean , T. H. O. Nguyen , and D. S. Layton , “Ferret Interferon (IFN)‐Inducible Transmembrane Proteins Are Upregulated by Both IFN‐α and Influenza Virus Infection,” Journal of Virology 95, no. 14 (2021): e0011121. 33952646 10.1128/JVI.00111-21 PMC8223922

[30] C. A. Coomer , K. Rahman , and A. A. Compton , “CD225 Proteins: A Family Portrait of Fusion Regulators,” Trends in Genetics 37, no. 5 (2021): 406–410. 33518406 10.1016/j.tig.2021.01.004

[31] J. J. Y. Chang , S. L. Grimley , B. M. Tran , et al., “Uncovering Strain‐ and Age‐Dependent Innate Immune Responses to SARS‐CoV‐2 Infection in Air‐Liquid‐Interface Cultured Nasal Epithelia,” IScience 27, no. 6 (2024): 110009, https://www.cell.com/iscience/abstract/S2589‐0042(24)01234‐3. 38868206 10.1016/j.isci.2024.110009 PMC11166695

[32] H. Jing , R. Tao , N. Dong , et al., “Nuclear Localization Signal in TRIM22 Is Essential for Inhibition of Type 2 Porcine Reproductive and Respiratory Syndrome Virus Replication in MARC‐145 Cells,” Virus Genes 55, no. 5 (2019): 660–672. 31375995 10.1007/s11262-019-01691-x PMC7089487

[33] J. Min , W. Liu , and J. Li , “Emerging Role of Interferon‐Induced Noncoding RNA in Innate Antiviral Immunity,” Viruses 14, no. 12 (2022): 2607. 36560611 10.3390/v14122607 PMC9780829

[34] D. Kim , J. Y. Lee , J. S. Yang , J. W. Kim , V. N. Kim , and H. Chang , “The Architecture of SARS‐CoV‐2 Transcriptome,” Cell 181, no. 4 (2020): 914, e10–921. 32330414 10.1016/j.cell.2020.04.011 PMC7179501

[35] J. Jia , W. Lu , B. Liu , et al., “An Atlas of Plant Full‐Length RNA Reveals Tissue‐Specific and Monocots‐Dicots Conserved Regulation of Poly(A) Tail Length,” Nature Plants 8, no. 9 (2022): 1118–1126. 35982302 10.1038/s41477-022-01224-9

[36] T. S. Zeng , D. S. Yang , A. A. Kelvin , and D. J. Kelvin , “Host Transcriptome Analysis of Ferret Tissues Following Henipavirus Infection,” in Nipah Virus, ed. A. N. Freiberg and B. Rockx , vol. 2682 (Methods in Molecular Biology, 2023), 281–299. 10.1007/978-1-0716-3283-3_20 37610589

[37] C. S. Grizer , K. C. Elliott , G. Singh , M. R. Vogt , and J. J. Mattapallil , “Respiratory Infection With Enterovirus D68 Induces Severe Acute Lung Inflammation in the Pediatric Ferret Model,” IScience 28, no. 8 (2025): 113071, https://www.cell.com/iscience/abstract/S2589‐0042(25)01332‐X. 40727933 10.1016/j.isci.2025.113071 PMC12302187

[38] E. S. Walsh , K. Yang , T. S. Tollison , et al., “Development of Ferret Immune Repertoire Reference Resources and Single‐Cell‐Based High‐Throughput Profiling Assays,” Journal of Virology 99, no. 4 (2025): e00181‐25. 40116504 10.1128/jvi.00181-25 PMC11998538

[39] S. A. Hardwick , W. Y. Chen , T. Wong , et al., “Spliced Synthetic Genes as Internal Controls in RNA Sequencing Experiments,” Nature Methods 13 (2016): 792–798, https://pubmed.ncbi.nlm.nih.gov/27502218/. 27502218 10.1038/nmeth.3958

[40] H. Li , B. Handsaker , A. Wysoker , et al., “The Sequence Alignment/Map Format and SAMtools,” Bioinformatics 25, no. 16 (2009): 2078–2079. 19505943 10.1093/bioinformatics/btp352 PMC2723002

[41] J. Schuster , M. E. Ritchie , and Q. Gouil , “Restrander: Rapid Orientation and Artefact Removal for Long‐Read cDNA Data,” NAR Genomics and Bioinformatics 5, no. 4 (2023): lqad108, 10.1093/nargab/lqad108. 38143957 PMC10748469

[42] H. Li , “Minimap2: Pairwise Alignment for Nucleotide Sequences,” Bioinformatics 34, no. 18 (2018): 3094–3100. 29750242 10.1093/bioinformatics/bty191 PMC6137996

[43] Y. Chen , A. Sim , Y. K. Wan , et al., “Context‐Aware Transcript Quantification From Long‐Read RNA‐Seq Data With Bambu,” Nature Methods 20, no. 8 (2023): 1187–1195. 37308696 10.1038/s41592-023-01908-w PMC10448944

[44] F. J. Pardo‐Palacios , A. Arzalluz‐Luque , L. Kondratova , et al., “SQANTI3: Curation of Long‐Read Transcriptomes for Accurate Identification of Known and Novel Isoforms,” Nature Methods 21, no. 5 (2024): 793–797. 38509328 10.1038/s41592-024-02229-2 PMC11093726

[45] G. M. Kurtzer , V. Sochat , and M. W. Bauer , “Singularity: Scientific Containers for Mobility of Compute,” PLoS One 12, no. 5 (2017): e0177459. 28494014 10.1371/journal.pone.0177459 PMC5426675

[46] J. T. Robinson , H. Thorvaldsdóttir , W. Winckler , et al., “Integrative Genomics Viewer,” Nature Biotechnology 29, no. 1 (2011): 24–26. 10.1038/nbt.1754 PMC3346182 21221095

[47] C. Y. Wan , J. Davis , M. Chauhan , et al., “IsoVis—A Webserver for Visualization and Annotation of Alternative RNA Isoforms,” Nucleic Acids Research 52, no. W1 (2024): W341–W347. 38709877 10.1093/nar/gkae343 PMC11223830

[48] M. I. Love , W. Huber , and S. Anders , “Moderated Estimation of Fold Change and Dispersion for RNA‐Seq Data With DESeq2,” Genome Biology 15, no. 12 (2014): 550. 25516281 10.1186/s13059-014-0550-8 PMC4302049

[49] A. D. Prjibelski , A. Mikheenko , A. Joglekar , et al., “Accurate Isoform Discovery With IsoQuant Using Long Reads,” Nature Biotechnology 41, no. 7 (2023): 915–918. 10.1038/s41587-022-01565-y PMC10344776 36593406

[50] A. Kuznetsova , P. B. Brockhoff , and R. H. B. Christensen , “lmerTest Package: Tests in Linear Mixed Effects Models,” Journal of Statistical Software 82, no. 13 (2017): 1–26.

[51] G. Yu , L. G. Wang , Y. Han , and Q. Y. He , “clusterProfiler: An R Package for Comparing Biological Themes Among Gene Clusters,” OMICS Journal of Integrative Biology 16, no. 5 (2012): 284–287. 22455463 10.1089/omi.2011.0118 PMC3339379

[52] Z. Du , H. Su , W. Wang , et al., “The trRosetta Server for Fast and Accurate Protein Structure Prediction,” Nature Protocols 16, no. 12 (2021): 5634–5651. 34759384 10.1038/s41596-021-00628-9

